# Colonic expression of *Ace2*, the SARS-CoV-2 entry receptor, is suppressed by commensal human microbiota

**DOI:** 10.1080/19490976.2021.1984105

**Published:** 2021-10-10

**Authors:** Adam Edwinson, Lu Yang, Jun Chen, Madhusudan Grover

**Affiliations:** a Division of Gastroenterology and Hepatology, Mayo Clinic, Rochester, MN, USA; b Department of Biomedical Statistics and Informatics, Mayo Clinic, Rochester, MN, USA

**Keywords:** Dysbiosis, coronavirus, germ-free mice, intestinal

## Abstract

Infection with severe acute respiratory syndrome-coronavirus-2 (SARS-CoV-2) is responsible for the COVID-19 pandemic. Angiotensin-converting enzyme 2 (*Ace2*) is expressed in the gastrointestinal (GI) tract and a receptor for SARS-CoV-2, making the GI tract a potential infection site. This study investigated the effects of commensal intestinal microbiota on colonic *Ace2* expression using a humanized mouse model. We found that colonic *Ace2* expression decreased significantly upon microbial colonization. Humanization with healthy volunteer or dysbiotic microbiota from irritable bowel syndrome (IBS) patients resulted in similar *Ace2* expression. Despite the differences in microbiota, no associations between α-diversity, β-diversity or individual taxa, and *Ace2* were noted post-humanization. These results highlight that commensal microbiota play a key role in regulating intestinal *Ace2* expression and the need to further examine the underlying mechanisms of this regulation.

The pandemic of COVID-19, caused by the severe acute respiratory syndrome-coronavirus-2 (SARS-CoV-2), has resulted in over 3 million deaths worldwide as of early 2021.^1^ The family of coronaviruses, which includes SARS-CoV-2, utilizes angiotensin-converting enzyme 2 (*Ace2*) as a receptor for viral attachment and intracellular entry.^2,3^ *Ace2* is expressed in a wide range of tissues including the liver,^4^ kidney, heart,^5^ lungs,^2^ and intestine,^6^ making each a potential route for viral entry and infection. A number of clinical studies have reported COVID-19 patients to have GI symptoms.^7–10^ Importantly, some studies have associated GI symptoms with disease severity, longer viral clearing, and poorer outcomes.^7,11–13^ Individuals with comorbidities such as obesity, diabetes, cardiovascular disease, and immune-compromised states, all of which have reported gut microbial dysbiosis,^14^ are at risk for severe COVID-19 symptoms.^15–18^ Additionally, gut microbiome diversity and composition in mice appears to be influenced by *Ace2* expression,^6^ and the microbiome can alter colonic *Ace2* expression in conventional animals.^19^ However, the effect of human microbiota on *Ace2* expression remains unknown. The intestinal microbiome may serve as an important determinant of COVID-19 predisposition and outcomes through its effects on *Ace2* expression.

We and others have shown dysbiosis in patients with irritable bowel syndrome (IBS).^20–22^ In this study, we examined the effect of commensal microbiota from healthy volunteers and IBS patients on the expression of *Ace2* in the colon using a humanized mouse model. Our goal was to understand how colonization with different microbial communities impacts *Ace2* expression and if specific bacterial taxa associate with colonic *Ace2* expression. We recruited Rome III IBS patients (n = 12, 11 females, age 42.4 ± 14.0) and healthy volunteers (n = 6, 5 females, age 48.7 ± 11.6) for collection of fecal samples and for obtaining sigmoid colonic biopsies. We used shotgun metagenomics to determine microbiota composition in these volunteers. Shotgun metagenomic sequences were analyzed using the SHOGUN v1.0.8 taxonomy profiler (BURST aligner).^23^ IBS patients had decreased microbial α-diversity (Inverse Simpson and Shannon indices, linear regression, *p*< .05, Figure 1a) and changes in microbiota composition compared to healthy controls (Bray–Curtis distance, PERMANOVA, *p*< .05, Figure 1b). Differential abundance analysis revealed that the phylum Euryarchaeota, the families *Odoribacteraceae, Methanobacteriaceae, Odoribacteraceae*, and *Sutterellaceae*, and the genus *Methanobrevibacter* were decreased in IBS patients, while Actinobacteria phylum was increased in IBS patients (permutation test,^24^ *FDR*<0.1, Benjamini–Hochberg procedure^25^).

**Figure 1.**
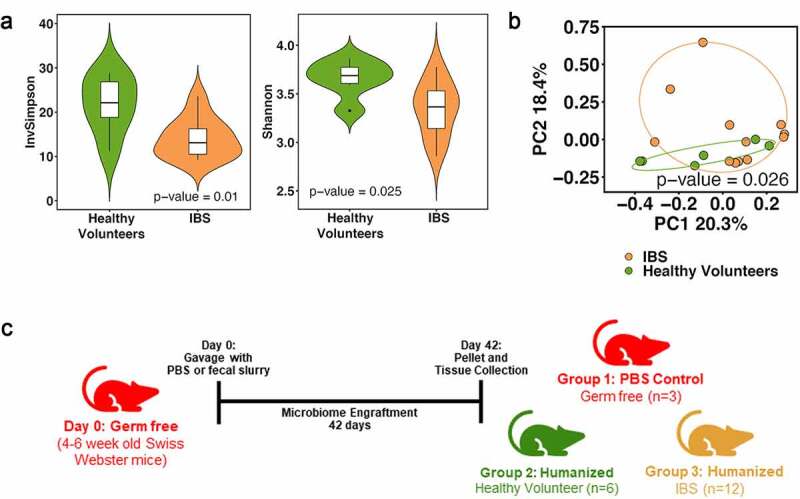
Fecal microbiota diversity and composition is different between IBS and healthy volunteers. (a) IBS patients have decreased α–diversity compared with healthy volunteers (InvSimpson and Shannon,*p*< .05). (b) PCoA plot of β-diversity shows IBS patients have differences in microbial composition compared to healthy volunteers. (c) Schematic for mouse humanization with healthy and IBS (dysbiotic) microbiota (n = 6–12 volunteers/group)

To determine how commensal human microbiota affects *Ace2* expression, we gave germ-free mice an oral gavage of fecal slurry (prepared anaerobically, 1:2 ratio of feces: pre-reduced PBS) from healthy volunteers (n = 6 volunteers). Mice were housed within flexible film isolators with access to both autoclaved food and water^20,26^ for 6 weeks to allow for microbiota to establish, after which fecal pellets and proximal colonic mucosal tissue were then collected (Figure 1c). Total RNA from mice and human colonic biopsies was sequenced and aligned using the Mayo Analysis Pipeline for RNA Sequencing (MAPRSeq v3.1.3) with the mouse genome reference mm10 and human genome reference hg38, respectively. We found that humanization resulted in a significant loss of *Ace2* expression in colonic mucosa compared to that of germ-free mice (333.4 ± 191.1 vs. 1914.4 ± 309.9 Fragments Per Kilobase of transcript per Million mapped reads (FPKM), *FDR*<0.001, Figure 2a). Furthermore, there was a 5.8-fold decrease in *Ace2* expression post-humanization (Figure 2b), indicating that human intestinal microbiota are able to suppress colonic *Ace2* expression.

**Figure 2.**
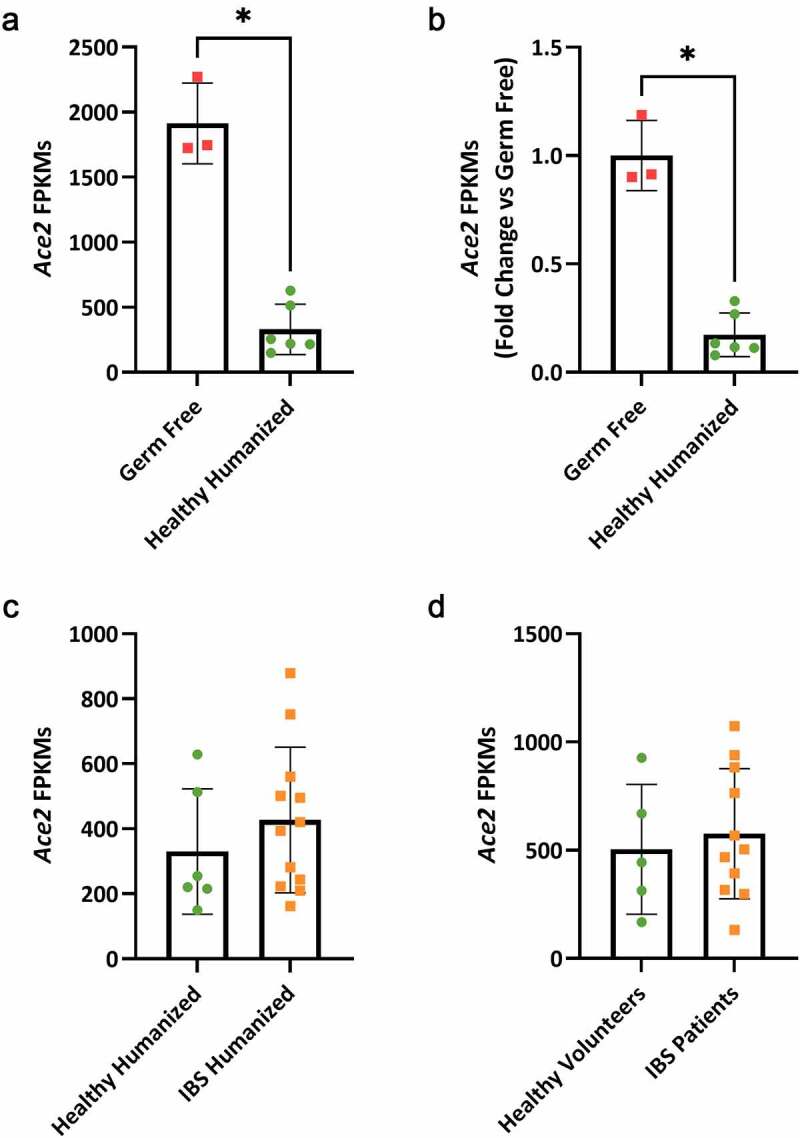
Colonic expression of *Ace2* in humanized mice and matched human donors. (a) Significantly lower colonic *Ace2* (333.4 ± 191.1 vs. 1914 4 ± 309.9) was seen in mice that were humanized with microbiota from healthy human donors compared to germ-free mice, *FDR*<0.001, n = 3–6 mice/group. (b) Humanized mice have a 5.8-fold lower *Ace2* expression compared to germ-free mice, Mann–Whitney, *p< .05, n = 3–6 mice/group. (c) Mice humanized with dysbiotic microbiota from IBS volunteers had similar *Ace2* expression as mice given healthy commensal microbiota, n = 6–12 mice/group. (d) *Ace2* expression in colonic biopsies from human healthy and IBS volunteers used for humanization was similar, n = 5–11 volunteers/group

We next wanted to understand if dysbiotic commensal microbiota from IBS patients would have a different effect on *Ace2* expression. We humanized mice using the same strategy (Figure 1c) and found no differences in *Ace2* expression post-humanization with healthy or IBS microbiota (Figure 2c). Additionally, no differences were noted between colonic *Ace2* expression of healthy volunteers and IBS patients (Figure 2d) suggesting IBS-associated microbial dysbiosis does not lead to changes in colonic *Ace2* expression in the GI tract. Compared to the mice humanized by the healthy microbiota, IBS microbiota humanized mice had a greater abundance of the phylum Firmicutes and the class Clostridia but lower abundance of the genus *Marvinbryantia* (permutation test, *FDR*<0.1). We next examined potential associations between microbial diversity or taxonomy and colonic *Ace2* expression. No significant associations were noted between α–diversity measures (Inverse Simpson and Shannon indices, linear regression, *p*> .1) and *Ace2* expression (Figure 3). Additionally, no significant association was found between β-diversity and the log transformed *Ace2* value while adjusting for disease status (Bray–Curtis distance, PERMANOVA *p*= .574). Finally, differential abundance analysis with *Ace2* expression did not identify any significant *Ace2*-associated taxa (permutation test, FDR > 0.1).

**Figure 3.**
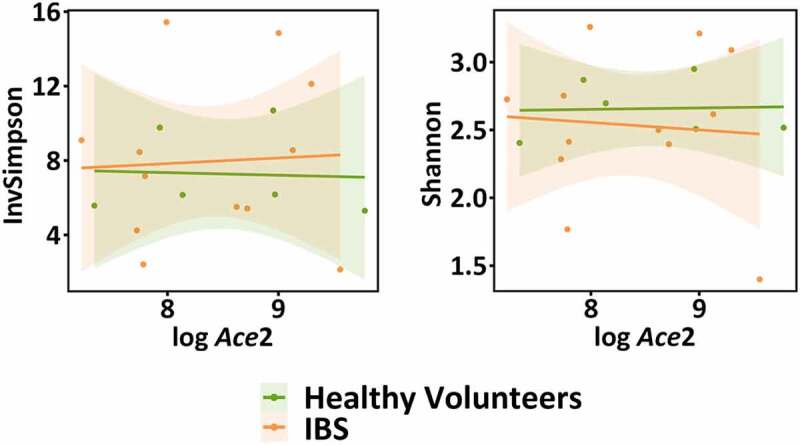
Associations between colonic *Ace*2 expression and microbiota of humanized mice. Linear modeling was used to test for associations between α–diversity of healthy and IBS microbiota with *Ace2* expression. No associations between α–diversity and FPKMs of *Ace2* in the colon were found (InvSimpson *p*= .492, Shannon*p*= .798), n= 6–12 mice/group

This study is one of the first to examine the role of human microbiota in regulating the expression of *Ace2* in the GI tract, describing a novel role for human commensal microbiota. Our humanized mouse model revealed that *Ace2* expression is significantly inhibited by both healthy commensal microbiota and dysbiotic microbiota from IBS patients. We also found similar *Ace2* expression in colonic biopsies from IBS patients and healthy individuals. It was recently shown that the mouse intestinal microbiome influenced *Ace2* expression in a wide range of organs and antibiotic treatment that depletes microbiota resulted in an increase in *Ace2* expression.^27^This is consistent with our observation of germ-free mice having significantly higher *Ace2* expression, which was suppressed after these mice were colonized with commensal human microbiota. Additionally, a recent study has shown that microbiota transplanted from *Ace2* knockout mice to germ-free animals resulted in severe colitis after dextran sulfate sodium challenge indicating an important relationship between the microbiome, *Ace2*, and intestinal homeostasis.^6^ The reduced levels of *Ace2* as a consequence of the intestinal microbiome therefore may have a protective role against SARS-CoV-2 infection by limiting potential receptors for viral entry via the colon. This is supported by single cell RNA sequencing data that has demonstrated expression of *Ace*2 by colonic epithelial cells is positively associated with viral entry into the cell.^28^ However, the mechanisms underlying microbial regulation of *Ace2* expression in the GI tract and its effect on SARS-CoV-2 entry into colonic epithelial cells need to be studied. Additionally, it still needs to be ascertained is if GI involvement by SARS-CoV-2 plays a role in the clinical course of COVID-19 or the associated GI manifestations of the disease.

Recently, the expression of *Ace2* has been shown to be significantly increased in individuals diagnosed with chronic obstructive pulmonary disease, smokers,^29^ hypertension, diabetes,^30,31^ and conditions associated with complications from COVID-19.^32–34^ A recent retrospective study demonstrated that among patients with functional GI disorders, diarrhea predominant IBS (IBS-D) was a positive predictor of COVID-19^35^ which may be explained by differences in the microbiota between the various subtypes of IBS.^20,36^ Interestingly, fecal metabolomics has also implicated intestinal microbiome as a predisposing factor for developing COVID-19.^37^ A study highlighted that COVID-19 patients have compositional differences in the microbiome structure that persist after the virus has cleared. The relative abundance of specific microbial taxa, specifically *Ruminococcus gnavus*,^37^ *Coprobacillus, Clostridium ramosum*, and *Clostridium hathewayi*^38^ correlated with increased disease severity, tissue damage, and immune response to the SARS-CoV-2 virus.^39,40^ It remains unclear, though, whether these changes are due to the inflammation or the therapies used to treat COVID-19.

In conclusion, we demonstrate an important role of commensal microbiota in regulating the expression of *Ace2* expression in the colon. Moreover, we provide evidence showing that the dysbiotic microbiota of IBS patients does not necessarily lead to dysregulated *Ace2*. The limitations of this study include small sample size as well as the examination of only one type of dysbiosis. It is possible that dysbiosis associated with other conditions such as obesity and diabetes confers different regulation of *Ace2* expression and increased risk for severe COVID-19. Future studies need to explore the role of commensal microbes on GI expression of *Ace2* which may affect predisposition for infection or poorer outcomes with SARS-CoV-2. Moreover, in patients, comorbidities, medications, and diet affect microbiota composition, reflecting the need for understanding the role of these factors as we explore if microbiota modulation can affect the course of SARS-CoV-2 infection.

Mayo Clinic Institutional Review Board approved all human studies and participants were also provided written, informed consent (IRB protocol: 12-006529; ClinicalTrials.gov identifier: NCT03266068). Animal experiments were approved by the Mayo Clinic Institutional Animal Care and Use Committee (Protocol #A00003420-18-R20). All data are displayed as means with standard deviation, with any frequencies and percentages for categorical variables. For all collected data, non-Gaussian distributions were assumed. Statistical tests were completed using a Mann–Whitney U test. When more than 2 groups were compared, a Kruskal-Wallis test (non-parametric one-way analysis of variance) was used. For all experiments and comparisons, a *p *< .05 was considered statistically significant.
